# Soldering and Bonding in Contemporary Electronic Device Packaging

**DOI:** 10.3390/ma18092015

**Published:** 2025-04-29

**Authors:** Yuxuan Li, Bei Pan, Zhenting Ge, Pengpeng Chen, Bo Bi, Xin Yi, Chaochao Wu, Ce Wang

**Affiliations:** 1 Nanjing Electronic Devices Institute, Nanjing 210000, China; panbei1981@126.com (B.P.); ge_zhenting@126.com (Z.G.); superjeson@126.com (P.C.); buaabb@163.com (B.B.); yixinxmy@163.com (X.Y.); 2 School of Mechanical Engineering and Automation, Fuzhou University, Fuzhou 350108, China; 3 State Key Laboratory of Precision Welding & Joining of Materials and Structures, Harbin Institute of Technology, Harbin 150001, China; cwanghit@hit.edu.cn

**Keywords:** electronic packaging, soldering, wafer bonding, die attachment, wire bonding, flip chip bonding

## Abstract

Electronic packaging can transform the chip to a device for assembly. Soldering and bonding are important procedures in the process of electronic packaging. The continuous development of packaging architecture has driven the emergence of improved soldering and bonding processes. At the same time, conventional soldering and bonding processes are still widely used in device packaging. This paper introduces two kinds of technologies in wafer bonding, direct and indirect, expounds on five kinds of die attachment processes, and also describes the process of ball bonding and wedge bonding in wire bonding in detail. Flip chip bonding and methods for making bumps are also described in depth. Bump bonding processes are vital for 3D-SiP packages, and the bonding technology of copper bumps is a research hotspot in the field of advanced packaging. The surface mount technology and sealing technology used in some electronic devices are also briefly introduced. This paper provides insights for researchers studying soldering and bonding in contemporary electronic device packaging.

## 1. Introduction

Integrated circuits (ICs) based on elemental semiconductors and compound semiconductors are the core of the current electronics industry. After the IC design is completed, wafers are produced in the cleanroom of a fab through various semiconductor manufacturing processes. Multiple chips are distributed on a wafer. The transistor is the main circuit element of the chips, and multiple chips can be obtained after dicing the wafer. Generally speaking, the wafer is extremely fragile, and the wafer will break with slight impact. Integrated circuits on the wafer cannot work directly in dust, humidity, salt fog, or a radiation environment. The pads on the wafer are tiny, which are not convenient for assembly operation. In addition, the integrated circuits will generate a lot of heat when working, and the wafer has no effective heat dissipation channel.

**Figure 1:**
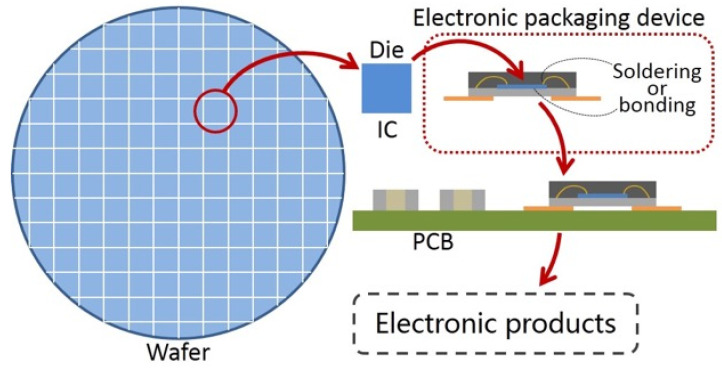
Schematic diagram that shows how a chip of wafer turns into an electronic product.

Electronic packaging can solve the above four problems [1,2]. Electronic packaging can provide mechanical support for the chip and protect the circuit elements on the chip. Electronic packaging enlarges the pads of the chip by creating terminals, which not only provide circuit connection, but also provide heat dissipation channels. Electronic packaging is an indispensable technology for semiconductor devices and microcircuit modules in engineering applications. Devices or modules are arranged on the PCBs of electronic products (mobile phones, computers, electronic bracelets, etc.), thus realizing the various functions of electronic products [3,4,5,6,7]. Figure 1 is a schematic diagram that shows how a chip of wafer turns into an electronic product.

**Figure 2:**
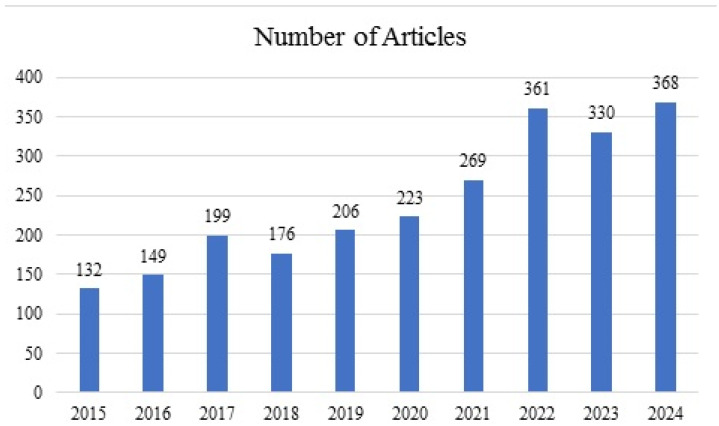
Growth in the number of publications on soldering or bonding in electronic device packaging from 2015 to 2024 (data sourced from the Web of Science database, statistics up to January 2025).

Soldering and bonding are important processes in electronic packaging. Based on the analysis of original data from the Web of Science database, recently, a growing trend in research on soldering or bonding in electronic device packaging can be observed, as shown in Figure 2.

The developing directions of electronic devices are miniaturization, low cost, 3D stacking, and enhanced heat dissipation [8,9,10]. Regarding specific processes, micro bump bonding [11,12,13,14], new material wire bonds [15,16,17], and enhanced heat dissipation materials [18,19] have been continuously developed. However, the conventional methods have not totally disappeared. Die attachment, wire bonding, and surface mounting technology (SMT) still have extensive applications in the electronics industry [20,21,22]. Furthermore, some processes in wafer manufacturing are gradually being integrated with the electronic packaging process, which considerably increases the circuit density and layout density of electronic devices [23,24]. A WLP (wafer level package) is a typical example. In the past, it was customary to divide the wafer into chips and then install the chips on the lead frame or substrate to realize the packaging. However, a WLP is completed on the wafer, which can greatly reduce the area of the package and improve the electrical performance of the device [25,26].

It is common in the industry to interpret the related technology of electronic packaging from the perspective of integrated circuits. From the view of material connection, the literature on electronic device packaging is relatively lacking. Based on the relationship between material connection and process progression, this paper divides the soldering and bonding in electronic device packaging into wafer bonding, die attachment, wire bonding, flip chip bonding and bump bonding, surface mounting technology, and sealing technology. There are many emerging technologies in the field of welding that have not been applied in semiconductor packaging [27,28,29]. By combing the current technologies, researchers can find the research direction of soldering technology in the semiconductor packaging industry.

## 2. Wafer Bonding

Wafer bonding refers to the process of connecting two clean and smooth wafer surfaces so that the two wafers can be combined and reach a certain strength (nearly 10~100 MPa) [1,23,30,31,32,33]. Stacking in the vertical direction of wafers can increase the density of transistors per unit area by times without breaking through the semiconductor manufacturing capacity. The fabrication of three-dimensional interconnected devices by wafer bonding is of great significance at a time when Moore’s law is getting closer and closer to the limit [30,34]. Micro-electro-mechanical systems (MEMS) not only need a three-dimensional structure, but also a sealed cavity. Wafer-level packaging has become an important technology of MEMS [26,34,35]. Furthermore, wafer bonding can also be used for heterogeneous integration [30,36].

Wafer bonding can be mainly divided into two categories: one is to treat the surface of the wafer and then realize the direct bonding between two wafers [31]; another is to realize the interconnection between wafers by means of adhesive, soldering material or surface metal. In wafer bonding, except for the interconnection by adhesive, which belongs to gluing, the other methods are essentially welding. Generally speaking, with the help of additional material layers, the bonding temperature can be reduced, and it is not easy to destroy the existing ion doping in the wafer. However, the introduction of additional material layers leads to the mismatch in thermal expansion coefficients, which may bring dangers such as warping or cracking in long-term use.

There are two main reliability risks of wafer bonding: one is warping or cracking caused by thermal stress, and the other is interface defects caused by lattice mismatch [1,32]. In recent years, there have been three main development trends of wafer bonding. First, the reliable bonding of various heterogeneous wafers [36]. The second is to reduce the implementation cost and process difficulty of wafer bonding such as bonding at low temperatures without additional material layers [31,33]. The third is to improve the state of the bonding interface to eliminate defects [32].

### 2.1. Direct Wafer Bonding

**Figure 3:**
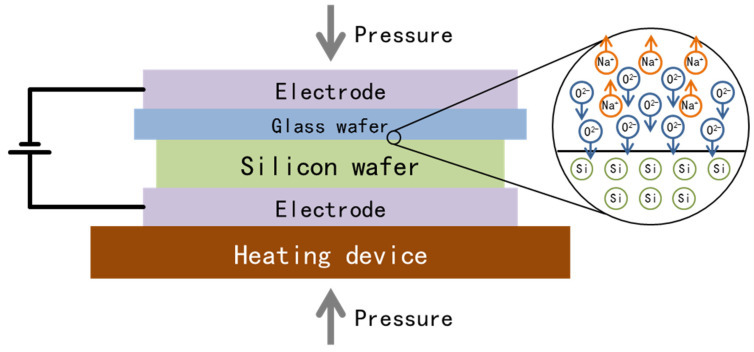
Schematic diagram of anodic bonding.

Anodic bonding is often used to bond a glass wafer to a silicon wafer [31,37]. As shown in Figure 3, a clean and flat silicon wafer and a glass wafer were contacted and heated, and an electric field was applied at the same time as pressure was exercised. Driven by an electric field of 300~1500 V, alkali ions enter the glass, and oxygen ions are enriched on the surface of the silicon wafer. Then, the silicon atoms on the surface are oxidized, thus realizing bonding.

There are other direct wafer bonding methods. The polished and clean silicon wafer is treated so that the surface of the wafer is covered with hydroxyl groups, and an annealing temperature that is at least 800 °C is needed to release water molecules and realize bonding. Finally, the two layers of silicon wafers are interconnected by oxygen atoms [30,31,33]. This is the initial direct bonding method, which also named wet hydrophilic bonding. In addition to wet hydrophilic bonding, there is wet hydrophobic bonding that produces a large number of Si-H bonds hanging on the surface of the silicon.

Because the temperature required by these methods is too high, low temperature plasma-activated direct bonding technology was developed [32,38]. The heat treatment temperature of low temperature plasma-activated direct bonding technology is 200~400 °C [31]. It is generally believed that after plasma treatment, the disordered structure is formed on the wafer surface, which leads to an increase in the removal rate of water molecules at the bonding interface, thus making it easier to form covalent bonds.

### 2.2. Indirect Wafer Bonding

GE Yuping [26] spined the adhesive based on non-photosensitive benzocyclobutene (BCB) on the glass wafer, and then pre-baked it at 90 °C for 60 s. Then, the non-photosensitive BCB was also deposited on the silicon wafer, and then the silicon wafer and glass wafer were aligned and contacted with the aid of a mask aligner. Finally, vacuum and pressure were applied for one hour at 250 °C to realize bonding through adhesive curing. After the bonding was completed, dicing was performed to obtain multiple devices, and the pads of the devices were on silicon or glass. The main advantage of wafer bonding with an adhesive is that the requirement for surface roughness is not high.

Metallurgical bonding can be achieved by soldering materials, and the bonding strength is usually higher than that of adhesives. Common eutectic bonding alloys are Au-Si, Al-Ge and Au-Sn [24,34]. Gold is electroplated on the silicon wafer in advance, and then another silicon wafer is contacted with the gold layer. The temperature of the Au-Si eutectic reaction is 363 °C. When the temperature is higher than 363 °C, gold and silicon will react chemically to produce a liquid eutectic with a specific atomic ratio (Au:Si = 81:19) that connects two wafers. Because the electroplated gold is relatively thin, the main body of the silicon wafer will not participate in the eutectic reaction. The drop in temperature or the change in atomic ratio caused by diffusion will transform this liquid phase into a solid phase, thus realizing the metallurgical bonding of two silicon wafers. Some studies have found superconducting properties in AuSn_4_ and SnSb [39,40], and it will be a remarkable breakthrough if the wafer bonding can create superconducting effect at the same time.

With the help of the same metal on the surface, metallurgical bonding can be achieved at a lower temperature without the participation of a liquid phase. This indirect wafer bonding is essentially diffusion bonding. Jia Ying-qian et al. [35] sputtered a gold layer on the clean silicon wafer surface, and then bonding was realized at a pressure of 6500 mbar and a temperature of 300 °C. In the process of bonding, gold and gold contact each other and deform locally, and then the pores are eliminated by atomic diffusion. In order to improve the efficiency of gold–gold bonding, the roughness of the gold layer on the surface needs to be small, so that the bonding between gold and gold will easily occur. It should be noted that if gold is directly sputtered onto the surface of silicon, the bond between gold and silicon may be weak. Normally Cr or Ti is needed as an intermediate layer between gold and silicon.

## 3. Die Attachment

After the wafer is sliced, a single chip with a complete circuit is called the die. Die attachment refers to the fixing of a single chip on a substrate. The upper surface of the die has rich transistors and some pads, and the lower surface is given to a die attachment material for fixing. The material for fixing the die must be strong enough and easy to employ. In most cases, it must be able to conduct electricity and heat [41]. There are many ways to fix the die on the package, substrate, or lead frame, for example, conductive adhesive bonding, nanosilver sintering, Au-Si eutectic bonding, soldering, and insulating adhesive bonding [19].

Die attachment has been used for a long time in the field of electronic device packaging [2,42]. There are two main reliability risks of new die attachment technologies. One is that the thermal expansion coefficients of the materials related to die attachment do not match, which leads to interface separation, and the other is silver migration under high temperature and high humidity [42,43,44]. There are three trends in the future development of die attachment: the first is to improve the electrical and thermal conductivity of die attachment materials [18,45], the second is to reduce the sintering temperature [2,46], and the third is to improve the storage stability of new die attachment materials [47].

### 3.1. Conductive Adhesive Bonding

At present, conductive adhesive bonding is the most common die attachment method. The conductive adhesive consists of a conductive filler, resin matrix, and additives for improving performance, where the conductive filler is mainly used to provide conductivity, the matrix resin is mainly to provide adhesion, and the additives are used to adjust the viscosity, curing conditions, and cured effect of the conductive adhesive [45,47]. Conductive fillers include silver-based fillers, copper-based fillers, carbon-based fillers, and composite conductive fillers, where silver fillers and composite conductive fillers are the most common [47]. Matrix resins include epoxy, polyurethane, and polyimide resins, and an epoxy resin matrix is the most widely used [45]. Conductive adhesives have the advantages of convenient use, simple process, and relatively low cost, but with the extension of time, the resin matrix can easily age.

The conductive adhesive is first coated on the surface to be bonded, then the die is placed on the conductive adhesive. During placement, a slight pressure is applied to ensure full contact between the die and the conductive adhesive. However, the pressure should not be too large, as if the pressure is too large, not only will the chip not bear it, but the conductive adhesive will also be squeezed out in large quantities. This is followed by heating and curing; curing make the matrix resin shrink and the conductive fillers contact each other, thus realizing electric conduction and heat conduction.

The research direction of conductive adhesives focuses on improving the electrical and thermal conductivity, reducing the cost, and improving the high temperature resistance and wet heat resistance [41,45,47].

### 3.2. Nanosilver Sintering

Compared with conductive adhesives, nanosilver sintering can provide better electrical and thermal conductivity, which is a good choice for power devices [18,48]. The essence of a conductive adhesive is that conductive fillers contact each other due to the curing shrinkage of the resin matrix. The sintering of nanosilver is realized by the diffusion and solidification of nanosilver particles.

**Figure 4:**
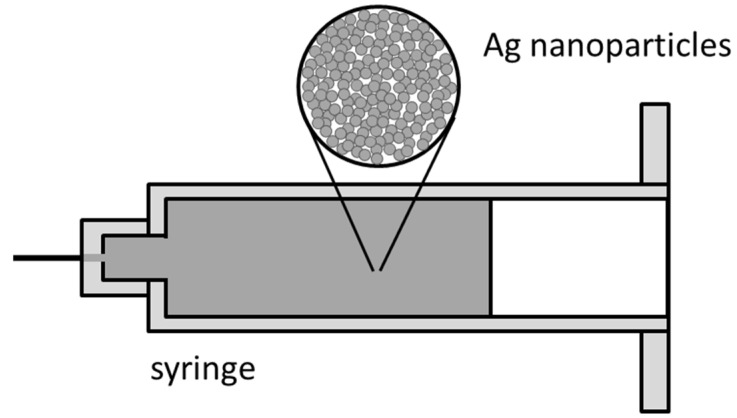
Nanoscale Ag paste.

Because nanosilver particles are tiny, the whole dispersion system of nanoscale Ag paste has a great surface area. Nanoscale Ag paste has a huge surface energy, and Ag nanoparticles cannot exist stably, especially in high temperature. When two particles make contact, a connection based on mutual diffusion will occur [48,49]. The combination of multiple particles can reduce the surface area, thus reducing the surface energy. Because of this, in order to prevent the silver nanoparticles from clustering or failure in room temperature, stabilizers are added to the silver paste [49]. The sintering of nanosilver paste needs heating (200 °C~300 °C), but it is usually unnecessary to apply pressure [18]. After heating, the surface of Ag nanoparticles can be activated. Surface diffusion mostly occurs in the initial stage of the sintering process, while the densification process mainly depends on the volume diffusion of silver. At the stage of volume diffusion, the pores shrink rapidly. Figure 4 shows a schematic diagram of the nanoscale Ag paste. At present, the sintering of nanocopper particles has also been verified, which provides a choice for lower-cost products [18].

### 3.3. Au-Si Eutectic Bonding

A single chip can be soldered onto the substrate by the eutectic bonding of gold and silicon. This basic principle is the same as that of Au-Si eutectic wafer bonding, which also uses the eutectic reaction between gold and silicon [20,41]. The silicon chip is placed on the surface of the substrate for heating, and there needs to be a gold coating with a thickness of more than 2 μm between the chip and the substrate. When the temperature is higher than 363 °C, the chip is pressed on the substrate and rubbed gently, and the gold and silicon undergo a metallurgical reaction to form a liquid eutectic. After the friction is stopped, the temperature drops, the eutectic turns into a solid state, and the chip and the substrate are combined. Au-Si eutectic bonding has strong electrical and thermal conductivity, good mechanical properties, and better reliability, although it has a high cost. Au-Si eutectic bonding is widely used in microwave hybrid integrated circuits and high reliability fields.

### 3.4. Soldering

The Au-Si eutectic reaction completes the die attachment, which is essentially soldering, but the chip itself needs to participate in the reaction. If it is not a silicon chip, it is impossible to complete Au-Si eutectic bonding with the help of its own silicon. In addition to Au-Si eutectic bonding, the chip can also be fixed by solders. Au80Sn20 (.wt%), which transforms into the liquid phase at 280 °C, and Sn96.5Ag3Cu0.5 (.wt%), which transforms into the liquid phase at 217 °C, are commonly used solders. First, the solder sheet is cut, then the solder sheet is placed between the chip and the surface to be soldered. The solder is melted by heating, then the two surfaces to be soldered are fully wetted with the liquid solder to form a metallurgical bond between them. After the temperature drops, the solder solidifies, and the die attachment is completed [19,50,51].

Whether it is Au80Sn20 or Sn96.5Ag3Cu0.5, the soldering temperature is relatively low, which brings convenience to the production process. However, it should be noted that the silicon chip or GaAs chip itself will not be directly metallurgically bonded with the solder. As a result, it is generally necessary to plate a layer of nickel and then a layer of gold on the surface of the chip to be soldered.

### 3.5. Insulation Adhesive Bonding

In the process of die attachment, a less insulating adhesive is used. The insulation adhesive needs to consider both the insulation and heat dissipation. Zhang Xiu-ju et al. [52] used epoxy resin E-44 as the matrix and increased the thermal conductivity of the adhesive by adding composite fillers such as silicon nitride particles and alumina particles. Dioctyl phthalate (DOP) was used as a plasticizer to adjust the viscosity and toughness of the adhesive. The thermal conductivity of the adhesive was 11.6 times that of the pure epoxy resin.

## 4. Wire Bonding

When the chip is fixed on the product through a die attachment process, it is necessary to establish an electrical connection between the chip and the outside circuits. Wire bonding is a widely used process to realize the chip electrical connection. In the process of chip manufacturing, the chip obtains pads, which are the circuit interfaces of the chip. The bonding pads on the chip are interconnected with the external bonding pads by wire bonding to realize the electrical connection [21,41]. Wire bonding can be divided into ball bonding and wedge bonding. Whether ball bonding or wedge bonding, ultrasonic thermo compression bonding is the mainstream method at present [53]. That is, heating, pressure, and ultrasonic waves are all needed in the bonding process. Commonly used wires are gold wire, silver wire, copper wire, and aluminum wire [54,55,56,57], where the diameter of the metal wire is from 15 μm to 500 μm [55].

Wire bonding is a very mature technology with relatively good operability and reliability [2,58]. At present, new progress mainly focuses on the development of new wires for wire bonding to balance the cost and performance [59], while the reliability challenge focuses on the corrosion inhibition, control of interfacial intermetallic compounds, and electromigration control brought by the new wires [57,59,60].

### 4.1. Ball Bonding

**Figure 5:**
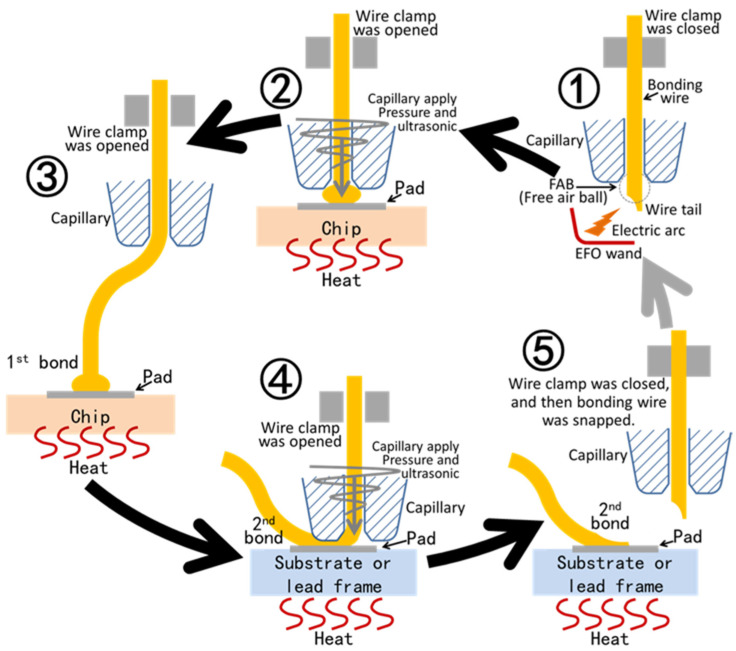
Schematic diagram of the ball bonding process.

Ball bonding refers to the method of wire bonding by burning the wire into a ball; however, the realization of ball bonding needs precision equipment. Ball bonding equipment includes a carrying device, a wire clamp, and a capillary. The carrying device transports and fixes the products to be bonded and heats the products. The wire passes through the wire clamp and then through the capillary. The wire clamp can control the movement of the wire, and when the clamp is closed, the wire cannot move. The whole ball bonding process is shown in Figure 5.

In the first step, a wire tail is exposed outside the capillary, and the exposed wire tail is instantly melted by the electric arc. After melting, the liquid metal forms a metal ball on the capillary due to surface tension, and this metal ball soon solidifies. At this time, the wire clamp is closed so that the wire can follow the capillary. In the second step, the capillary presses the metal ball on the chip pad, the wire clamp is opened, and the equipment applies ultrasonic waves to the capillary. The ultrasonic waves make the possible oxides on the pad surface and the metal wire surface leave the bond and expose the atoms inside the pad and the metal ball. The bond energy of the inside atoms is not saturated. Because of the temperature and pressure, the atoms of the bonding pad and the metal ball diffuse with each other, and bonding is realized after the ultrasonic wave stops. At this time, the bonding between the metal wire and the first pad is completed, and the original metal balls will basically flatten. In the third step, the wire clamp is opened, and then the capillary is transferred to the top of the second pad. Because the wire clamp is in a relaxed state and the end of the wire has been bonded, the wire will slip out of the capillary. In the fourth step, the capillary directly presses down on the second pad, and then ultrasonic waves are applied. In the same way, bonding is realized after the ultrasonic wave stops. In the fifth step, the wire clamp is closed, the capillary moves outward, and the gold wire is pulled off at the bonded position. The equipment continues to bond, that is, repeat the above five steps.

Because the capillary of ball bonding is perpendicular to the bonding pad, the second bonding pad in ball bonding can be located at any position of the first bonding pad. There is no need to adjust the direction in the process of ball bonding, which makes the speed of ball bonding faster. Because the balls have to be melted to form, it is difficult to realize the wire bonding of oxidizable metals such as aluminum by ball bonding. In addition, due to the existence of a ball at the first bonding pad, the spacing between two parallel loops is larger.

### 4.2. Wedge Bonding

**Figure 6:**
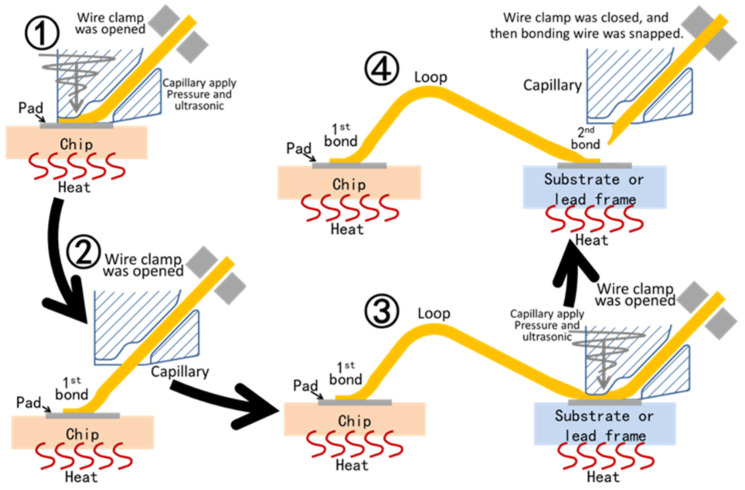
Schematic diagram of the wedge bonding process.

Wedge bonding equipment also has a carrying device, a wire clamp, and a capillary. However, the capillary of wedge bonding is greatly different from that of ball bonding, and the metal wire in wedge bonding does not pass through the capillary along the center of the capillary. The whole process of wedge bonding is shown in Figure 6.

In the first step, the wire clamp is closed, so that the wire can follow the capillary. Then, the capillary presses the end of the metal wire onto the chip pad, and the wire clamp is opened. The equipment applies ultrasonic waves to the capillary. The ultrasonic waves make the possible oxides on the pad surface and the metal wire surface leave the bonding surface and expose the atoms inside the pad and the metal wire contact area. The bonding energy of the inside atoms is not saturated. Because of the temperature and pressure, the atoms in the bonding pad and the metal wire contact area diffuse with each other, and bonding is realized after the ultrasonic wave stops. At this time, the bonding between the metal wire and the first pad is completed. In the second step, the capillary can only move backward and upward, and then move over the second pad. Because the wire clamp is in a relaxed state and the end of the wire has been bonded, the wire will slide out from the capillary. Thirdly, the capillary directly presses down on the second pad, and then the ultrasonic wave is applied. In the same way, bonding is realized after the ultrasonic wave stops. In the fourth step, the clamp is closed, the capillary moves outward, and the metal wire is pulled off at the bonded position. The equipment continues to bond, that is, repeat the above four steps.

**Figure 7:**
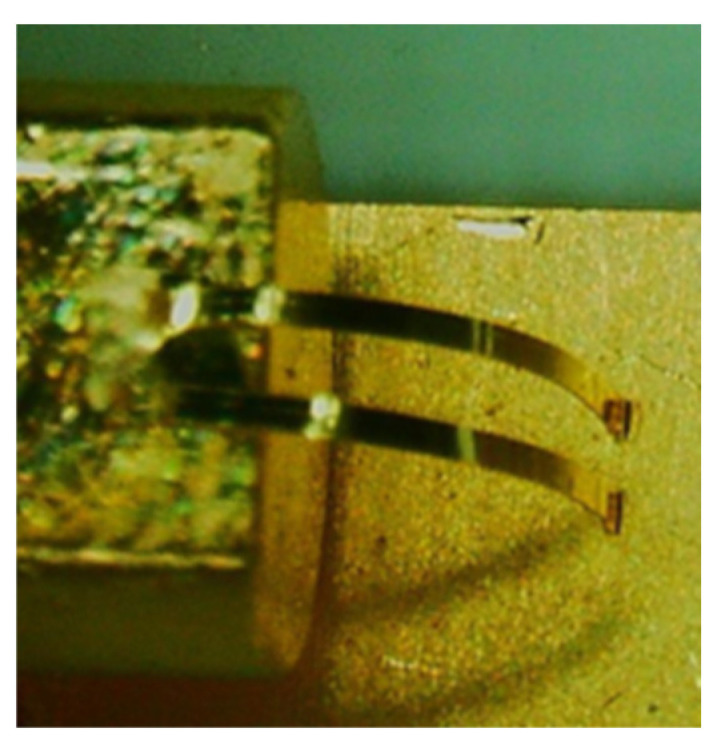
Gold strips bonded by wedge bonding.

Because of the unique threading method of wedge bonding, the second bond in wedge bonding can only be located behind the first bond. It is often necessary to adjust the direction when bonding wires at various positions, so the bonding speed of wedge bonding is slow. Wedge bonding does not need to melt the metal, thus aluminum wire wedge bonding and copper wire wedge bonding are more common. The height of the wedge bonding loop can be lower, and the spacing between two wedge bonding loops can also be smaller. Wedge bonding can also bond the strip. Figure 7 shows the bonding of a gold strip by wedge bonding.

### 4.3. Challenges of New Wire Bonding

A major research direction of wire bonding is to reduce the cost by adopting wires made of new materials. It is generally believed that most electronic devices that use gold wire at present may switch to silver wire or copper wire [15,16]. However, low-cost metal wires will bring challenges in reliability. Corrosion inhibition, the control of interfacial intermetallic compounds, and the electromigration control of copper wire and silver wire are the current research hotspots [54,57,59,61]. These challenges can be solved by adjusting the alloy composition of the metal wire, adjusting the microstructure of the metal wire and optimizing the wire bonding process [15,16,17,21,54,57,59,61].

## 5. Flip Chip Bonding and Bump Bonding

**Figure 8:**
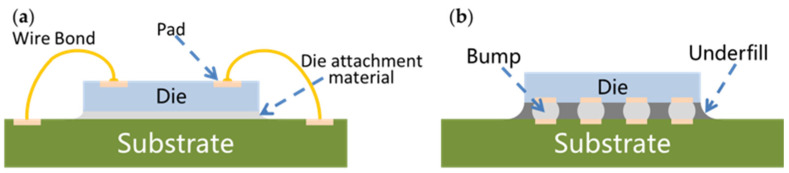
Schematic diagrams of a normal chip and flip chip. (**a**) Normal chip; (**b**) flip chip.

Die bonds can fix the chip, and wire bonds can electrically interconnect the chip with the outside circuits. However, the metal wires are fanned out, which increases the area of the package. At the same time, the length of the metal wire is relatively long, and the frequency characteristic is poor. Making bumps on the pads of the chip, then turning the chip upside down and bonding with the bumps facing down is called flip chip bonding [10,62,63]. Flip chip bonding not only realizes the fixation of the chip, but also realizes the electrical interconnection between the chip and the outside circuits through one-time bonding. This not only improves the production efficiency and reduces the area of the package, but also reduces the parasitic inductance. The schematic diagrams of a normal chip and flip chip are shown in Figure 8.

The traditional way is to flip the chip on the package substrate. In the current advanced packaging, in order to improve the integration and production efficiency, there are three new application scenarios of the flip chip, namely, die to die (D2D), die to wafer (D2W), and wafer to wafer (W2W). D2D refers to flipping one or more chips on another chip to form a whole, and then using it. D2W refers to flipping many chips on another wafer, and then scribing the new wafer for use. W2W refers to flipping one wafer onto another to form a stacked wafer. Advanced packaging based on the flip chip is the basis of CoWoS (chip-on-wafer-on-substrate) technology, Intel EMIB (embedded muti-die interconnect bridge) technology, and Foveros technology [10].

**Figure 9:**
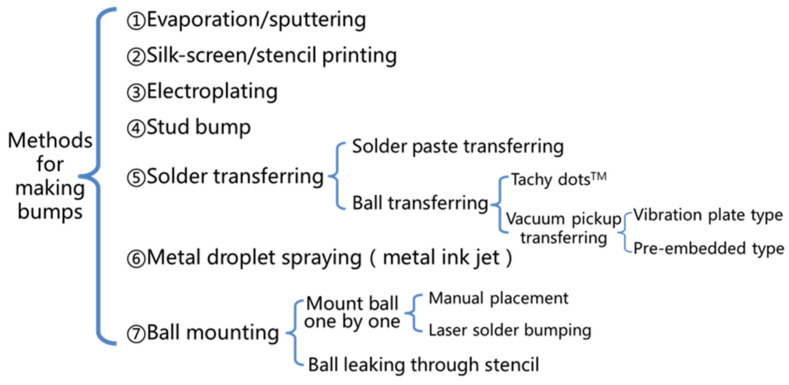
Methods for making bumps.

Generally speaking, a flip chip includes four key technologies, namely UBM (under bump metallurgy) preparation technology, bump preparation technology, flip chip bonding technology, and underfill technology [64,65]. UBM refers to metallization in the chip electrical connection area during the wafer manufacturing stage to provide bonding pads for chip bumps. The bonding pad of the chip in wire bonding can also be regarded as UBM. Generally, bump making is carried out in the wafer state, and there are various bump making methods that are developing with the development of packaging. Bump making methods mainly include evaporation/sputtering, silk-screen/stencil printing, electroplating, the stud bump method, solder transferring, metal droplet spraying, and ball mounting [10,63,66]. The classification of methods for making bumps is shown in Figure 9.

**Figure 10:**
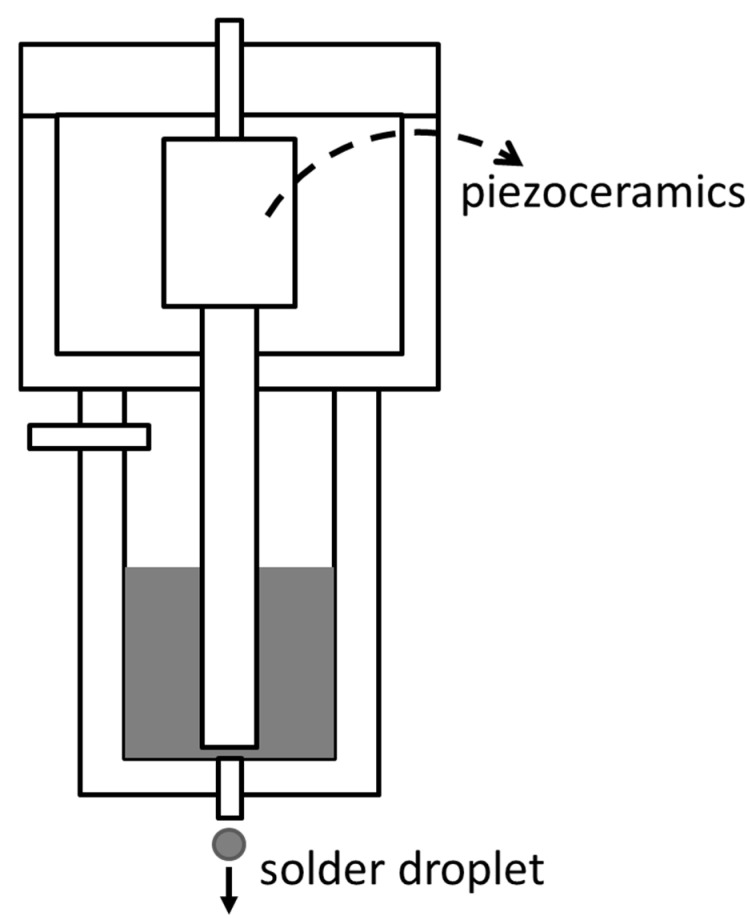
Conceptual view of the solder droplet jetting system.

The evaporation/sputtering deposition method refers to making bumps in specific areas by evaporation or sputtering. Common methods include magnetron sputtering, laser evaporation, and electron beam evaporation [10,66,67]. The silk-screen/stencil printing method is often used to make solder bumps, that is, the solder paste is printed in a specific area with the help of the screen, and the solder paste becomes solder bumps after reflow. The electroplating method refers to electroplating in a specific area, and areas that do not need electroplating can be shielded. Copper pillars are usually made by the electroplating method [68]. The principle of the stud bump method is the same as that of ball bonding. After the FAB is formed, the FAB is pressed on the pad, and then the wire is broken. Solder transferring needs a special sucker and fixture, and the specific position of the bump is realized by the sucker and fixture. This method is mostly used for solder ball arrays. Metal droplet spraying is a new method to prepare bumps. By spraying a certain volume of solder droplets to the pads, the bump is made, and the production efficiency is obviously improved [67]. Figure 10 is a conceptual view of the solder droplet jetting system. A typical representative of ball mounting is laser ball bumping one by one. The laser is aimed at the pad, and then the solder ball falls in the optical path, and the solder ball is “soldered” onto the pad by laser heating [69].

**Figure 11:**
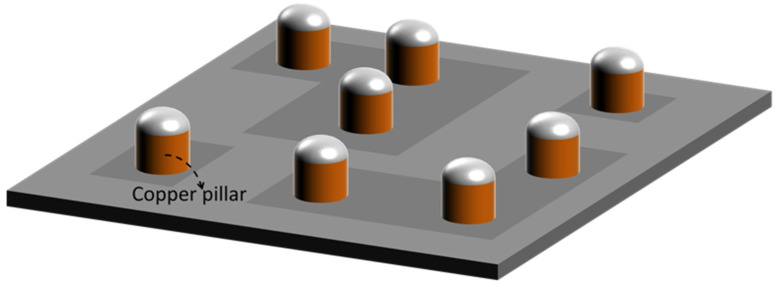
Copper pillar.

Flip chip bonding technology is the key in the flip chip process. Flip chip bonding mainly includes ultrasonic thermo compression bonding, reflow soldering, and thermal compression bonding [14]. The mechanism of ultrasonic thermo compression bonding is similar to the ball bonding of wire bonding. The chip with gold bumps on the front side is sucked from the back by a suction nozzle, and then the chip is pressed on the substrate. By applying heat, ultrasonic waves, and pressure, the combination of the gold bumps and the substrate pads is realized. Ultrasonic thermo compression bonding has low requirements for equipment. Gold bump bonding can be realized by ball bonding equipment, so the technical difficulty is relatively low. Flip chip bonding based on reflow soldering is suitable for solder bumps. The chip with solder bumps on the front side is sucked from the back by the suction nozzle, then the solder bumps on the chip are dipped in flux, and finally, the chip is placed on the corresponding pads of the substrate. Solder bumps undergo melting and re-solidification in the process of reflow soldering, thus realizing the connection between the chip and the substrate. Hot-pressed flip chip bonding is mostly suitable for copper pillar bumps. A copper pillar, such as that in Figure 11, is plated on the UBM layer of the chip by electroplating, and the end of the copper pillar may or may not have a low melting point solder such as SAC305. Bumps of the pure copper pillar can better realize the high-density bump array connection [9]. At present, the bonding of pure copper columns is a hot research topic, and many ways are being adopted in the industry to improve the diffusion rate of copper atoms. At the same time, efforts are being made to reduce the temperature and pressure of hot-pressing flip chip bonding [9,70,71,72]. Low temperature and low pressure can adapt to many chips, and a reliable copper–copper connection below 250 °C can be realized at present [11,12]. This is of great significance for 3D-SiP. However, further lowering the interconnection temperature can be compatible with more solder and cover more temperature-sensitive chips, so it still needs to be tackled continuously.

Although the application of copper bumps has obvious advantages in realizing miniaturization and a high density of packaging, the intermetallic compound (IMC) layer is the key challenge for copper bump bonding [9,72,73]. IMC layers with different structures have far-reaching effects on heat transfer, electromigration, and long-term reliability [74]. A thick and continuous bad IMC layer will lead to a significant decrease in the heat dissipation, conductivity, and structural strength of the package. In severe cases, cracks may directly originate [72]. Nanomaterials have great application space in improving the reliability of copper–copper bonding [75]. Some studies have confirmed that polycrystalline Cu/Sn composite nanoarrays can regulate the growth of IMCs [72,73]. Using specific nano twin copper as the UBM material can inhibit the formation of Kirkendall voids and the dissolution of a UBM layer under the action of high temperature electromigration [76].

Considering that the thermal expansion coefficients of chip materials, bumps, and package substrates are quite different, after flip chip bonding, the bump areas are often underfilled in order to reduce the damage caused by thermal stress or mechanical impact [64]. All of the bumps are wrapped by the liquid underfill driven by surface tension, and the gas between the bumps is squeezed out. The underfill can be cured at a low temperature (less than 150 °C), and can then bear loads.

## 6. Surface Mounting Technology

Surface mounting technology (SMT) is a quite mature electronic component assembly technology [22,77,78]. The reliability evaluation of lead-free solder has basically been completed by the industry [79,80]. The deformation ability of lead-free solder at room temperature is not as good as that of lead solder, but they actually have their own advantages and disadvantages in terms of reliability [7,78,81]. With the exception of electronic components working with a high power or high load, through-hole insertion technology is also adopted, and other electronic components are arranged on printed circuit board (PCB) by SMT [3,78]. Although SMT is always used on PCBs, it is still possible to be used in electronic device packaging. For example, in radar RF devices (modules), resistors, capacitors, and dies are integrated on the same substrate and then packaged into one device [82]. At this time, the chip can be “die bond + wire bond” or flip chip bonding, and surface mount components such as resistors and capacitors can be surface mounted. Conventional processes of SMT include solder paste distribution, electron component placement, reflow soldering, and automatic optical inspection (AOI) [5,22,77]. First, solder paste (a mixture of flux and solder powders) is distributed on the pad by screen printing or dispensing. Then, the electron components are placed through the mounter, and the pins of the electron components are in contact with the solder paste. In the process of reflow soldering, the solder powders in the solder paste melt and gather, and at the same time, they are metallurgically bonded with the pins and pads of the substrate. After the temperature returns to room temperature, all of the pins are soldered with the substrate pads, thus realizing the integration of all electronic components into the one circuit. After the soldering is completed, the AOI will inspect solders to confirm that the soldering quality meets the requirements. In a few highly reliable fields, the residual flux should be cleaned. SMT is also developing toward miniaturization and high density. For example, the smallest external dimension of resistors and capacitors for surface mounting is 0.4 mm × 0.2 mm (01005). Beyond that, there are more and more defects in SMT that cannot be inspected by visible light. AXI (automatic X-ray inspection) is used to inspect the defects where light cannot reach [83].

## 7. Sealing Technology

**Figure 12:**
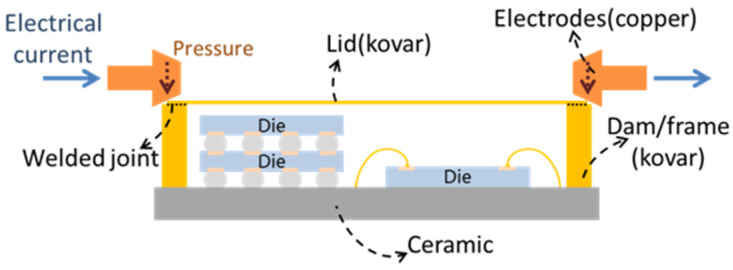
Illustration of the parallel seam welding process.

For most electronic devices, chips are encapsulated by a plastic encapsulating material, and then the package is molded. The technology of encapsulation is very mature and does not involve soldering or bonding between materials. However, there are still many products in which the sealing of packages is realized by soldering or bonding the cover plate such as cermet packaging devices and MEMS packaging devices. Common methods include parallel seam welding, soldering, laser welding, ultrasonic welding, and gluing [4,84]. Parallel seam welding is a kind of resistance welding, and the two to be welded must conduct electricity. A metal frame (generally made of kovar) is designed on the periphery of the substrate where the chip is arranged, and then the metal cover plate is placed on the frame. The electrode is energized by pressure, and joule heat is generated by the contact resistance between two objects, and the edges of the two objects are melted to realize welding [85,86,87]. Figure 12 is an illustration of the parallel seam welding process. Soldering sealing refers to melting the solder, metallurgical bonding between the liquid solder and the two, and sealing after solidification. Commonly used solders include Au80Sn20 and Sn-Ag-Cu series solders. Laser welding is divided into laser brazing and laser fusion welding. Laser brazing refers to the use of laser melting solder to achieve sealing, which is essentially a kind of brazing. Laser fusion welding refers to the use of lasers to melt the edge of the cover plate and the shell so that the two are mixed together in the liquid phase, and welding is realized after cooling. The application of ultrasonic welding is limited, and it is mainly used for welding between thermoplastics. The gluing method is easy to operate and can be applied to metals, ceramics, and glass, so it has a wide range of uses. The most commonly used adhesive for the cover plate is a thermosetting adhesive, usually a thermosetting epoxy [84].

## 8. Comparison

In electronic device packaging, different soldering and bonding technologies have different applications. Although wafer bonding and micro bump bonding are being more and more used in advanced packaging, there is no obvious intergenerational substitution relationship between wafer bonding, die attachment, wire bonding, surface mounting technology, flip chip bonding, and bump bonding. In order to obtain the best market competitiveness, there are often many kinds of bonding modes in 3D-SiP modules. Table 1 compares four commonly used technologies.

## 9. Conclusions

(1) With the continuous promotion of high density and low cost, new soldering and bonding processes are constantly emerging in electronic devices. At the same time, conventional die attachment and wire bonding are still widely used. New technologies, such as low temperature wafer bonding or micro bump bonding, are more complementary to the traditional technologies than the substitutes.
(2) The process of the semiconductor wafer level is increasingly integrated with the process of the electronic packaging level. Wafer bonding is directly used in electronic device packaging, and flip chip bonding is also used on wafers. The integration of semiconductor manufacturing and packaging obviously improves the production efficiency, but the diversification of structure also brings hidden dangers of reliability. These risks need to be identified through more accurate multi-physical field simulations and in-depth interface analyses.
(3) The innovation of die attachment mainly focuses on developing new adhesive materials to improve the electrical and thermal conductivity of the chip and at the same time reduce the sintering temperature and cost. The low-temperature sintering of highly reliable nanoscale Ag paste and low-cost nanoscale Cu paste is a research hotspot. However, at the same time, it is necessary to solve the storage stability before sintering and the long-term reliability after sintering.
(4) At present, the development of wire bonds mainly focuses on developing new alloy wires to improve the mechanical reliability and reduce the cost. Copper wire and silver wire will eventually be connected with pads made of different materials, and the control and evaluation of interfacial intermetallic compounds will be the key. In addition, the bonding process of copper wire needs to be continuously optimized to adapt to various application scenarios.
(5) As an extension of flip chip bonding technology, micro bump and micro bump bonding technology are vigorously being developed. The related technologies of precision welding and 3D printing have great application potential in the manufacture of micro bumps. Various forms of copper–copper bonding are the research focus at present, and copper–copper bonding has been applied in advanced packaging. The reliability of copper–copper bonding is affected by the IMC layer, and the IMC layer can be controlled by prefabricating nanomaterials in order to improve the service performance.

## Figures and Tables

**Table 1:** Comparison of different soldering and bonding technologies.

	Wafer Bonding	Die Attachment	Wire Bonding	Flip Chip Bonding and Bump Bonding
Electrical conductivity	Very high	High	Low	Average
Heat dissipation	Very high	High	Low	Average
Cost	Very high	Average	Low	High
Integration density	Very high	Average	Low	High
Utilization	Large-area bonding between two wafers	Fixing a chip on a substrate or a lead frame	Electrical connection between chip pads and the outside	Electrical connection between chip pads and the outside
Reliability challenges	① Warpage or crack caused by thermal stress ② Interface defects caused by lattice mismatch	① Separation caused by thermal stress ② Silver migration under high temperature and high humidity	① Corrosion and IMC caused by new wire	① Brittle (IMC) layer of interface ② Thermal stress
Development tendency	① Reliable bonding of heterogeneous wafers ② Reduce the implementation cost and process difficulty	① Higher electrical and thermal conductivity ② Lower sintering Temperature ③ Higher storage Stability	① Developing new wire for wire bonding	① Smaller size bump bonding ② Bonding techniques for micro bumps with diverse nanomaterials

## Data Availability

No new data were created or analyzed in this study.
